# Characterisation of betalain biosynthesis in *Parakeelya* flowers identifies the key biosynthetic gene DOD as belonging to an expanded LigB gene family that is conserved in betalain-producing species

**DOI:** 10.3389/fpls.2015.00499

**Published:** 2015-07-07

**Authors:** Hsiao-Hang Chung, Kathy E. Schwinn, Hanh M. Ngo, David H. Lewis, Baxter Massey, Kate E. Calcott, Ross Crowhurst, Daryl C. Joyce, Kevin S. Gould, Kevin M. Davies, Dion K. Harrison

**Affiliations:** ^1^Centre for Native Floriculture, School of Agriculture and Food Sciences, The University of Queensland, GattonQLD, Australia; ^2^New Zealand Institute for Plant & Food Research LimitedPalmerston North, New Zealand; ^3^Victoria University of WellingtonWellington, New Zealand; ^4^New Zealand Institute for Plant & Food Research LimitedAuckland, New Zealand

**Keywords:** dioxygenases, DODA, LigB, betalain, flower color, *Parakeelya*, pigmentation, Caryophyllales

## Abstract

Plant betalain pigments are intriguing because they are restricted to the Caryophyllales and are mutually exclusive with the more common anthocyanins. However, betalain biosynthesis is poorly understood compared to that of anthocyanins. In this study, betalain production and betalain-related genes were characterized in *Parakeelya mirabilis* (Montiaceae). RT-PCR and transcriptomics identified three sequences related to the key biosynthetic enzyme Dopa 4,5-dioxgenase (DOD). In addition to a LigB gene similar to that of non-Caryophyllales species (Class I genes), two other *P. mirabilis* LigB genes were found (*DOD* and *DOD-like*, termed Class II). *PmDOD* and *PmDOD-like* had 70% amino acid identity. Only *PmDOD* was implicated in betalain synthesis based on transient assays of enzyme activity and correlation of transcript abundance to spatio-temporal betalain accumulation. The role of *PmDOD-like* remains unknown. The striking pigment patterning of the flowers was due to distinct zones of red betacyanin and yellow betaxanthin production. The major betacyanin was the unglycosylated betanidin rather than the commonly found glycosides, an occurrence for which there are a few previous reports. The white petal zones lacked pigment but had DOD activity suggesting alternate regulation of the pathway in this tissue. *DOD* and *DOD-like* sequences were also identified in other betalain-producing species but not in examples of anthocyanin-producing Caryophyllales or non-Caryophyllales species. A Class I *LigB* sequence from the anthocyanin-producing Caryophyllaceae species *Dianthus superbus* and two *DOD-like* sequences from the Amaranthaceae species *Beta vulgaris* and *Ptilotus* spp. did not show DOD activity in the transient assay. The additional sequences suggests that DOD is part of a larger LigB gene family in betalain-producing Caryophyllales taxa, and the tandem genomic arrangement of two of the three *B. vulgaris* LigB genes suggests the involvement of duplication in the gene family evolution.

## Introduction

Betalains, comprised of the red–violet betacyanins and yellow–orange betaxanthins, are plant pigments of special interest with regard to both their evolutionary origin and possible roles in stress-tolerance. They have a limited taxonomic distribution, having been found only in the flowering plant order Caryophyllales (Clement et al., 1994) and a few genera of the fungal phylum Basidiomycota. Betalains and the more common anthocyanin pigments have never been found together in the same plant (Mabry et al., 1963; Kimler et al., 1970), although betalains and other types of flavonoids do co-occur (Giannasi, 1988). For this reason, it is presumed that betalains, particularly the betacyanins, perform the same functions as anthocyanins, e.g., facilitating pollination and seed dispersal, and ameliorating abiotic stresses.

Although there has long been scientific interest in betalain pigments, there is much that remains to be elucidated. Notably, the evolution of this pathway and the mechanism behind the mutual exclusivity with anthocyanins has not been resolved (Sakuta, 2014). Intriguingly, there are a few families (Caryophyllaceae and Molluginaceae) within the core Caryophyllales that produce the more common, and extensively studied, anthocyanin pigments. However, as neither family are basal to the betalain families, and Molluginaceae actually resides within the betalain lineages, the betalain families are not monophyletic (Cuenoud et al., 2002; Brockington et al., 2009) and there is a complex evolutionary history of pigmentation in the order (Brockington et al., 2011). The biosynthetic and regulatory pathway for betalain production has also not been fully characterized. Until recently, only one of the enzymes responsible for formation of the colored compounds had been characterized at the molecular genetic level, dopa 4,5-dioxygenase (DOD). However, recent breakthroughs for *Beta vulgaris* (beet) have identified a gene for a second biosynthetic step (Hatlestad et al., 2012) and the first regulatory gene for the pathway, an R2R3MYB (Davies, 2015; Hatlestad et al., 2015).

**FIGURE 1:**
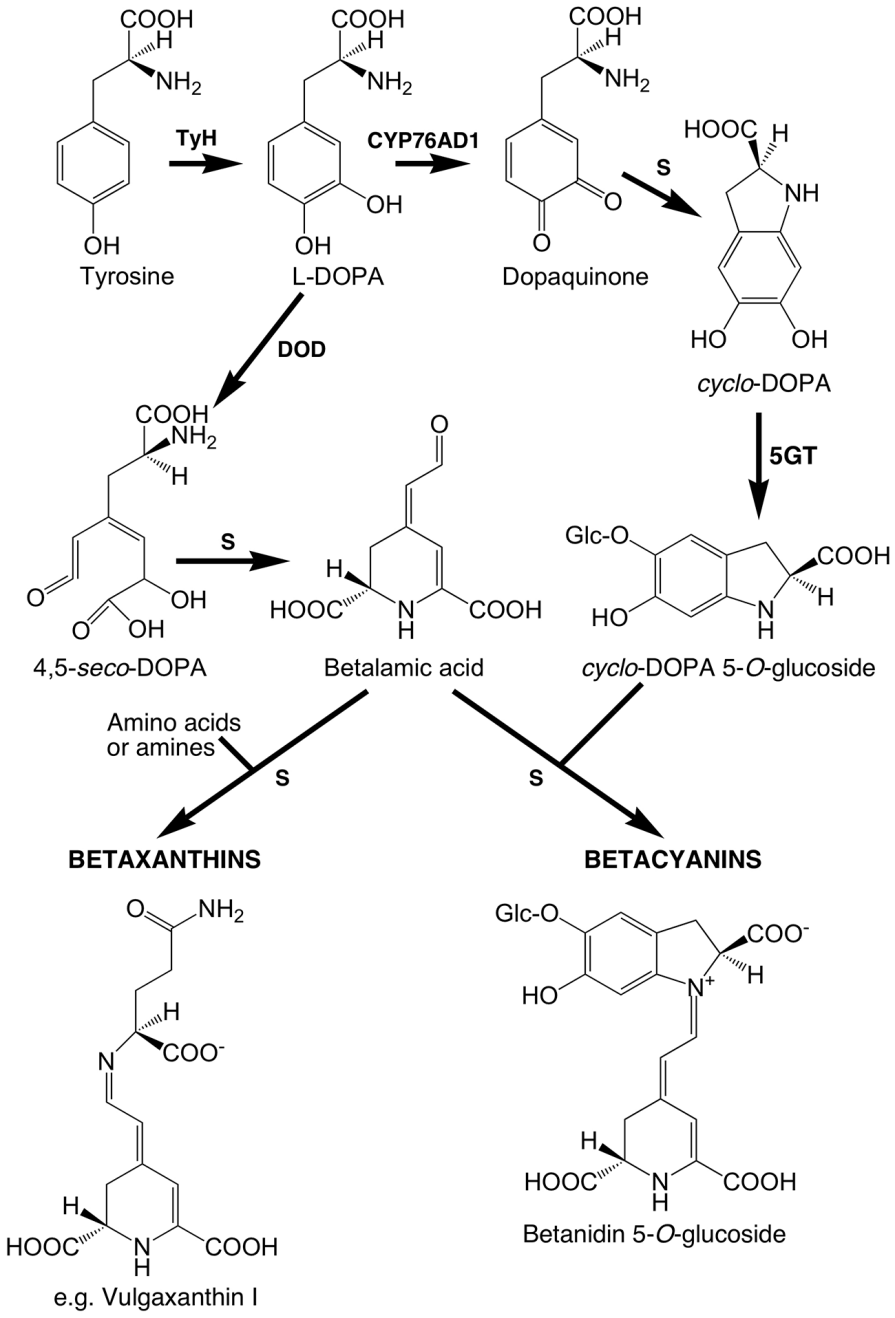
**Putative core betalain biosynthetic pathway in plants.** S, spontaneous reaction; the primary enzymatic reactions are tyrosine hydroxylating activity (TyH), the enzyme for which has not been unequivocally determined; CYP76AD1, a cytochrome P450 enzyme that catalyzes oxidation of L-DOPA for the formation of *cyclo*-DOPA; and DOPA 4,5-dioxygenase (DOD) catalyzing the cleavage of L-DOPA needed for the formation of betalamic acid. Betacyanins normally accumulate as glycosides and may also be acylated. Regarding glucosylation at the 5-*O*-position, it appears that it is primarily done by *cyclo*-DOPA 5-*O*-glucosyltransferase (5GT) prior to the conjugation that forms the betacyanin.

In contrast to the phenylalanine-derived anthocyanins/flavonoids, betalains are derived from tyrosine. Betalamic acid (BA) is the chromophore of all plant betalains. In betalain biosynthesis (**Figure 1**), tyrosine is hydroxylated to form 3,4 dihydroxyphenylalanine (DOPA). A 4,5 aromatic ring cleavage of DOPA is then required to form BA, which proceeds through an unstable 4,5-*seco*-DOPA intermediate that spontaneously rearranges to form BA (Fischer and Dreiding, 1972; Schliemann et al., 1998). DOPA is also oxidized to *O*-DOPA-quinone, which spontaneously rearranges to form *cyclo*-DOPA, the key moiety that determines betacyanin formation. The conjugation reactions that form the pigments are thought to occur spontaneously (Strack et al., 2003; Harris et al., 2012) and involve *cyclo*-DOPA (or its glycoside) and BA for betacyanin synthesis, and BA and an amino acid or amine derivative other than *cyclo*-DOPA for betaxanthins. Thus, only three primary enzymatic reactions are thought to be involved in the pathway from tyrosine to the first colored compounds. Betalains are stored in the vacuole and betacyanins are normally stored as glycosides, with glucosylation possible at either the betanidin or *cyclo*-DOPA steps of the biosynthetic pathway (Strack et al., 2003; Sakuta, 2014).

The biosynthetic enzymes for the latter two core steps have been characterized at the molecular level; DOD, which catalyzes DOPA ring cleavage, and CYP76AD(1), which forms *cyclo*-DOPA. CYP76AD has so far only been characterized from *B. vulgaris* (CYP76AD1, Hatlestad et al., 2012), *Mirabilis jalapa* (CYP76AD3, Suzuki et al., 2014) and *Amaranthus hypochondriacus* (CYP76AD1, Casique-Arroyo et al., 2014), but DOD has been studied across a few species. Gene sequences for DOD were first identified from the betalain producing fungus *Amanita muscaria* (Hinz et al., 1997) and subsequently in plants from moss rose (*Portulaca grandiflora*; Christinet et al., 2004). Plant DOD has homology to the LigB domain of bacterial extradiol 4,5-dioxygenases, while the fungal DOD belongs to a distinct group of dioxygenases (Christinet et al., 2004). Despite their divergent sequences and distinct catalytic activity, both the plant and fungal DOD enzymes can function when transiently expressed in anthocyanin-producing species, as demonstrated by the associated accumulation of betalains when DOPA is supplied (Harris et al., 2012). LigB homologs have been identified in a wide range of plant orders (Christinet et al., 2004). However, those from non-betalain orders are readily distinguished by a distinctive sequence motif in the proposed catalytic domain (Christinet et al., 2004; Bahramnejad et al., 2010), and the *Arabidopsis* LigB (the only one for which a function has been assigned) is involved in the production of compounds not related to betalains, substituted α-pyrones derived from phenylalanine (Weng et al., 2012). On this basis, we hereafter refer to them as Class I and Class II (containing DOD) type LigB homologs, respectively. The only report of a sequence for a Class I type LigB from a betalain-producing species is for *Amaranthus hypochondriacus* (*AhDODA-2*), which is referred to by the authors as being close to ‘DODA-like’ genes of anthocyanin-producing species (Casique-Arroyo et al., 2014).

While there is a great deal of information on the regulation of the expression of anthocyanin biosynthetic genes, there is little similar information for the betalain pathway. Although there are some studies showing *DOD* transcript levels correlating to betalain levels this seems to be variable between species (Wang et al., 2007; Casique-Arroyo et al., 2014), and studies on a wider range of species are required (Sakuta, 2014). In this study we examine betalain biosynthesis in a member of the Montiaceae family, *Parakeelya mirabilis*, by detailing betalain production, LigB Class II gene expression patterns and enzyme activities, and by generating a transcriptome database for the species. *Parakeelya* resides within the core Caryophyllales and its members (39–55 morphologically diverse species) are endemic to Australia (Carolin, 1993; Hershkovitz, 1998; Obbens, 2006). As a member of the Montiaceae, it is phylogenetically well separated from the model species *B. vulgaris*, which resides in the Amaranthaceae. Among the significant findings is the identification of two *P. mirabilis* LigB genes with distinct enzymatic activities and transcript abundance patterns, and we therefore also examine the occurrence and enzymatic activity of similar LigB genes from other members of the Caryophyllales. The results suggest previously unidentified LigB enzyme activities within the Caryophyllales, and also establish *Parakeelya* as a useful new model for studies to elucidate the betalain biosynthetic pathway.

## Materials and Methods

### Plant Material

An endemic Australian accession of *P. mirabilis*, supplied by Nindethana seed service (Albany, WA, Australia) was collected from Gascoyne, WA, Australia (25°02′586^′′^S, 115°12′336^′′^E). The genus was formed as a separation from the *Calandrinia* genus of the Portulacaceae family (Nyffeler and Eggli, 2010), and the species was formally known as *Calandrinia mirabilis*. The lines were propagated by tissue culture as in Wickramasinghe et al. (2009). After 3 weeks, tissue culture plants were transplanted into potting medium of composted pine bark:sawdust:sand (2:4:1 v/v). The plants were grown in a greenhouse under ambient light conditions with drip irrigation at The University of Queensland, Gatton, QLD, Australia (27°33′272^′′^S, 152°20′154^′′^E). The average temperature and relative humidity (RH) were 25 ± 7°C (range: 12–45°C) and 70 ± 29% RH (range: 12–100%), respectively. Three genotypes (95-2, 146-2, and 149-1) were used as three biological replicates throughout the study. Floral developmental was divided into; stage 1 (closed bud approximately 0.5 cm long, no pigmentation), stage 2 (closed bud <1.0 cm long, pigmentation starting), stage 3 (closed bud <1.5 cm long, pale pigmentation), stage 4 (bud length <2.0 cm, strong pigmentation, petals extend from sepals), and stage 5 (bud length <3.0 cm, fully pigmented, open flowers). Stage 5 flowers were used for HPLC-LCMS analysis, mRNA extraction for transcriptome sequencing, and total RNA extraction for gene expression analysis of different plant tissues (including violet, white, red and yellow sectors of petals, stamens, stigmata, ovaries and leaves). Stage 4 flowers were used for gene cloning.

### Betalain Pigment Analyses

High-performance liquid chromatography as in Harris et al. (2012), with HPLC gradients as per Kugler et al. (2007), was used for betalain pigment identification and quantification in the different color sectors of *P. mirabilis* petals and the pigmented tissue from *PmDOD* transient expression experiments, with additional structural identification of pigments in the petal samples by LC-MS. Fresh tissue, or freeze dried tissue resuspensed in 200 μL deionised water (Svenson et al., 2008), was extracted in 80% (v/v) aqueous methanol containing 50 mM sodium ascorbate as in Schliemann et al. (1999). Peaks on the HPLC chromatograms were assessed against retention time and spectral maxima for known betalain pigments in a beetroot control sample, and further analyzed by LC-MS. The LC-MS system consisted of a Thermo Electron Corporation (San Jose, CA, USA) Finnigan Surveyor MS pump, Finnigan MicroAS auto-sampler, Finnigan Surveyor PDA detector and a Phenomenex ThermaSphere TS-130 column heater. The same mobile phase and elution gradients as for HPLC were applied. Two μL of extract was separated by reverse phase chromatography using a 4 mm × 2 mm 10 μm Aqua guard cartridge and a 250 mm × 2.1 mm 4 μm Phenomenex Synergi Hydro-RP C18 column at 25°C with a flow rate of 200 μL min^-1^. The eluent was scanned by PDA at 200–700 nm and analyzed by API-MS (Thermo-Finnigan LTQ, 2D linear ion-trap) with electrospray ionization (ESI, positive). Data were acquired for parent masses from m/z 250 to 1500 amu with MS^4^ fragmentation. Data were processed with the aid of Xcalibar^®^2.05 (Thermo-Electron Corporation).

A spectrophotometric method was used to measure total betalain content in *P. mirabilis* tissues. Fresh plant tissue (∼100 mg) was homogenized to a fine powder in liquid nitrogen and extracted with 2 mL 80% (v/v) aqueous methanol with 50 mM ascorbic acid (Schliemann et al., 1999). After centrifugation at 15,000 × *g* for 30 min at 4°C, the supernatant was quantified using a Pharmacia LKB-UltrospecIII UV/Vis spectrophotometer (Pharmacia LKB, Cambridge, UK). The aqueous pigment extracts were diluted with McIlvaine buffer (pH 6.0, citrate–phosphate) to obtain absorption values around 1.0 at absorption maxima. The betalain content (*BC*) was calculated after Kugler et al. (2004, 2007): [*BC* (mg kg^-1^) = (*A* – *A_650_*) *× DF × MW × V_e_ × 1000/(𝜀 × L × W_d_)*]; where, *A* is absorbance value at the absorption maximum corrected by subtraction for non-specific absorbance at 650 nm, *DF* is the dilution factor, *L* is the cuvette path length (1 cm), *V_e_* is total extract volume (mL), and *W_d_* is dry weight of extracted material. Betacyanin were quantified using the MW (550 g mol^-1^) and molar extinction coefficient (𝜀 = 60,000 L mol^-1^ cm^-1^ in water at max 538 nm) of betanin. Betaxanthins were quantified using the MW (339 g mol^-1^) and molar extinction coefficient (𝜀 = 48,000 L mol^-1^ cm^-1^ in water at max 470 nm) of vulgaxanthin I. For conversion of betalain content from fresh weight (FW) to dry weight (DW), a different set of fresh tissue samples were collected and freeze dried for 1 week until completely dry. The weights of samples were measured before and after freeze-drying. The FW/DW ratio for three biological replicates was calculated and used for conversion.

### Isolation of cDNAs and Vector Construction

Based on homologous gene sequences obtained from publicly available databases, degenerate primers (Supplementary Table S1) were designed to amplify fragments of *LigB* homologs, *elongation factor 1α* (*EF1α*) and *actin* (*ACT*) from cDNA derived from *P. mirabilis* stage 4 petal RNA. Total RNA was extracted using an Isolate Plant RNA Mini Kit (Bioline, UK) and treated with DNase I (Promega, USA). First strand cDNA was made using a cDNA Synthesis Kit (Bioline, UK) with the dT_18_ primers. The rest of the coding sequences were obtained by standard 3′- and 5′-rapid amplification of cDNA ends (RACE) polymerase chain reaction (PCR).

All vectors were based on pART7 (Gleave, 1992), which has a multiple restriction enzyme cloning site between the *Cauliflower Mosaic Virus 35S* (*35S*) promoter and terminator of the *Octopine Synthase* gene. For *PmDOD1, PmDOD-like*, and *PhybDOD-like* the cDNAs amplified started at the putative translation start sites and included part of the 3′ untranslated regions (3′ UTR). This amplified *PmDOD-like* had two amino acid changes [open reading frame (ORF) E192Q and T265S] compared to the original *PmDOD-like* sequence. *PhybDOD-like* was isolated from green leaf tissue of cv. Little Princess, a hybrid between *P. exaltatus* var. *semilanatus* B110A and *P. nobilis*. *BvDODA* was synthesized (GenScript USA Inc.) based on the ORF sequence from GenBank accession AJ583017. Synthesis incorporated restriction endonuclease sites flanking the putative translation start and stop codons to allow cloning into pART7. For *BvDODA1*, an ORF cDNA including stop codon was isolated from Swiss chard using primers specific for the GenBank sequence HQ656027. This cDNA was ligated into pGEMT-Easy and the *Eco*RI fragment then taken for vector construction. Plasmid preparations for transient assays used the AxyPrep^TM^ Plasmid Midiprep Kit (Axygen Biosciences). Primer sequences are given in Supplementary Table S1.

### Transient Gene Expression Assay

The assay used *Antirrhinum majus* line AG110 petal tissue and *Allium cepa* (onion) scales. *A. majus* line AG110 is a *nivea* mutant lacking chalcone synthase activity, and therefore has white flowers because no anthocyanins or other flavonoids are produced. The adaxial epidermis of each petal lobe sample was bombarded. Each experiment used three dorsal petal lobe sets per DNA/gold preparation, and each experiment was conducted at least twice. Each experiment compared a *DOD-like* construct with a *DOD* construct using the same biolistic conditions, with the latter also functioning as a positive control. Tissue surface sterilization, tissue culture and particle bombardment were as described in Shang et al. (2007), except for using a vacuum of -90 kPa, a final construct DNA concentration of 1.0 or 1.4 μg DNA per 1.0 mg gold particles, and 4 or 5 μL of gold suspension per shot. Each tissue sample was shot twice. The internal control to demonstrate transformation efficiency was pRT99GFP co-precipitated with the construct of interest at 0.4 μg DNA per 1.0 mg gold particles. After incubation overnight the bombarded tissues were vacuum infiltrated with sterilized water or either 10 or 1 mM L-DOPA (Sigma-Aldrich, USA), blotted (in some cases after a rinse with water to remove surface DOPA), and placed back on the medium for incubation overnight. Tissue was observed under white and blue (505–550 nm) light for the presence of pigments and fluorescence, respectively. Images were recorded using either an Olympus SZX fluorescent microscope (Olympus Corp., Tokyo, Japan) with a Leica DC500 digital camera (Leica Camera AG, Solms, Germany) or a Leica M205FA microscope with a Leica DFC550 digital camera. The assay using white onion scale was performed as for *A. majus*, except that surface sterilization was for 2 min and the solution did not contain Tween, the distance between the gene gun column and tissue was 12 cm, 1/2 MS medium was used with 1.2% (w/v) agar and 3% (w/v) sucrose, and tissue was incubated under 40 μmol m^-2^ s^-1^ light from Polylux 36 W fluorescent tubes (Polylux XL, Hungary) at room temperature. Tissue was observed under an Olympus BH2 light microscope (Olympus, Japan) and images recorded using a Canon IXUS400 digital camera (Tokyo, Japan).

### Quantitative Real-Time PCR (qPCR)

TaqMan primers and minor groove binder (MGB) probes labeled with 6-carboxy fluorescein (6FAM^TM^) are given in Supplementary Table S1. RNA extraction, DNase I treatment and cDNA synthesis were carried out as given already for isolation of cDNAs except that random hexamers were used as primers for reverse transcription. The total RNA used in each reverse transcription reaction was 500 ng. Quantitative (q)PCR was performed in a total reaction volume of 25 μL (25 ng cDNA sample, 1x TaqMan^®^Universal PCR Master Mix (Life Technologies, Australia), 300 nM of each gene specific primer and 200 nM MGB probe) using an ABI7000 thermocycler (Applied Biosystems). Amplification was performed using the parameters of 2 min at 50°C and 10 min at 95°C, followed by 40 cycles of 15 s at 95°C and 30 s at 60°C. The cycle threshold (Ct) value for each reaction was calculated. Expression of the reference genes, *EF1α* and *ACT*, was consistent across development, with CT values changing by less than 2.0. Three biological replicates and three technical replicates for each biological replicate were performed. The natural logarithm transformed data were used for analysis. Expression of *PmDOD1* and *PmDOD2* was normalized by the BestKeeper Index of *EF1α* and *ACT* using BestKeeper^©^ Software version 1 (Pfaﬄ et al., 2004).

### Maximum Likelihood Analysis

The ORF DNA from the LigB homologs were aligned using Muscle (Edgar, 2004) followed by Maximum Likelihood analysis using the PhyML program (Guindon and Gascuel, 2003) within the bioinformatics software Geneious version 6.1.6 (Biomatters, Auckland, New Zealand). The default parameters of substitution model set to LG, proportion of variable sites set to fixed 0, number of substitution rate categories set to 4, gamma distribution parameter set to estimated, optimize set to topology/length/rate were used. Topology search used either NNI (Default, fast) or BEST (Best of NNI and SPR search). Bootstrap analysis with 1000 replicates was used to estimate the confidence of each tree node.

### Transcript Sequencing and Assembly

Violet and yellow petal sectors were dissected, mRNA was isolated using the μMACS mRNA Isolation Kit (Miltenyi Biotec), and sent to the Australian Genome Research Facility (Brisbane, QLD, Australia) for 454 GS-FLX Titanium sequencing. *De novo* assembly of FASTA sequence was performed using MIRA3 (Chevreux et al., 1999) using default settings. Violet and yellow petal assemblies used 211,574 and 203,929 reads, respectively, with average read length of 321 and 323 bases, respectively. The yellow petal *de novo* assembly yielded 21,189 transcripts containing 11,943,110 bases with mean length of 563 bases, a maximum transcript length of 5,009 bases, and an assembly N50 of 603 bases. The violet petal *de novo* assembly yielded 22,662 transcripts containing 13,139,842 bases with mean length of 579 bases, a maximum transcript length of 8,136 bases and an assembly N50 of 620 bases. Data is available on the NCBI database linked to the Biosample accession numbers SAMN03769446 and SAMN03769447 for the violet and yellow samples, respectively.

## Results

### Betalain Biosynthesis in *Parakeelya*

**FIGURE 2:**
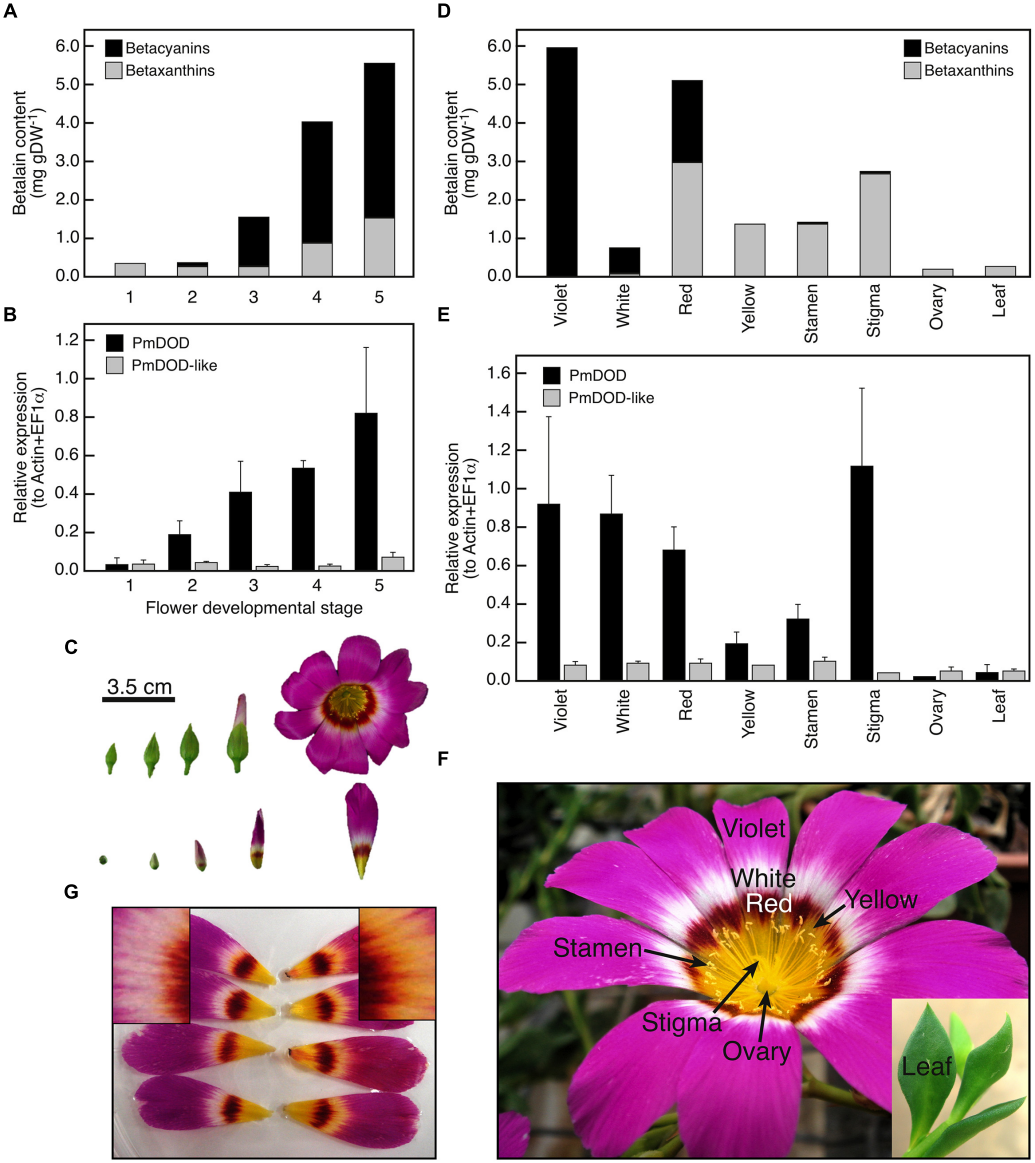
**Spatio-temporal patterns of betalain accumulation and *DOD* and *DOD-like* expression in *Parakeelya mirabilis*.** Accumulation **(A)** and expression **(B)** in petals during flower development **(C)**, and accumulation **(D)** and expression **(E)** in different colored petal sectors (developmental stage 5) and other organs **(F)**. Relative expression was determined by qPCR and normalized to the geometric mean of *EF1α* and *ACT* expression. SE for three biological replicates is shown. **(G)** Shows petals fed a control solution (left) or a solution containing DOPA (right). The inserts show a magnification of the white petal region, with yellow pigmentation apparent in the DOPA but not control fed petals.

**FIGURE 3:**
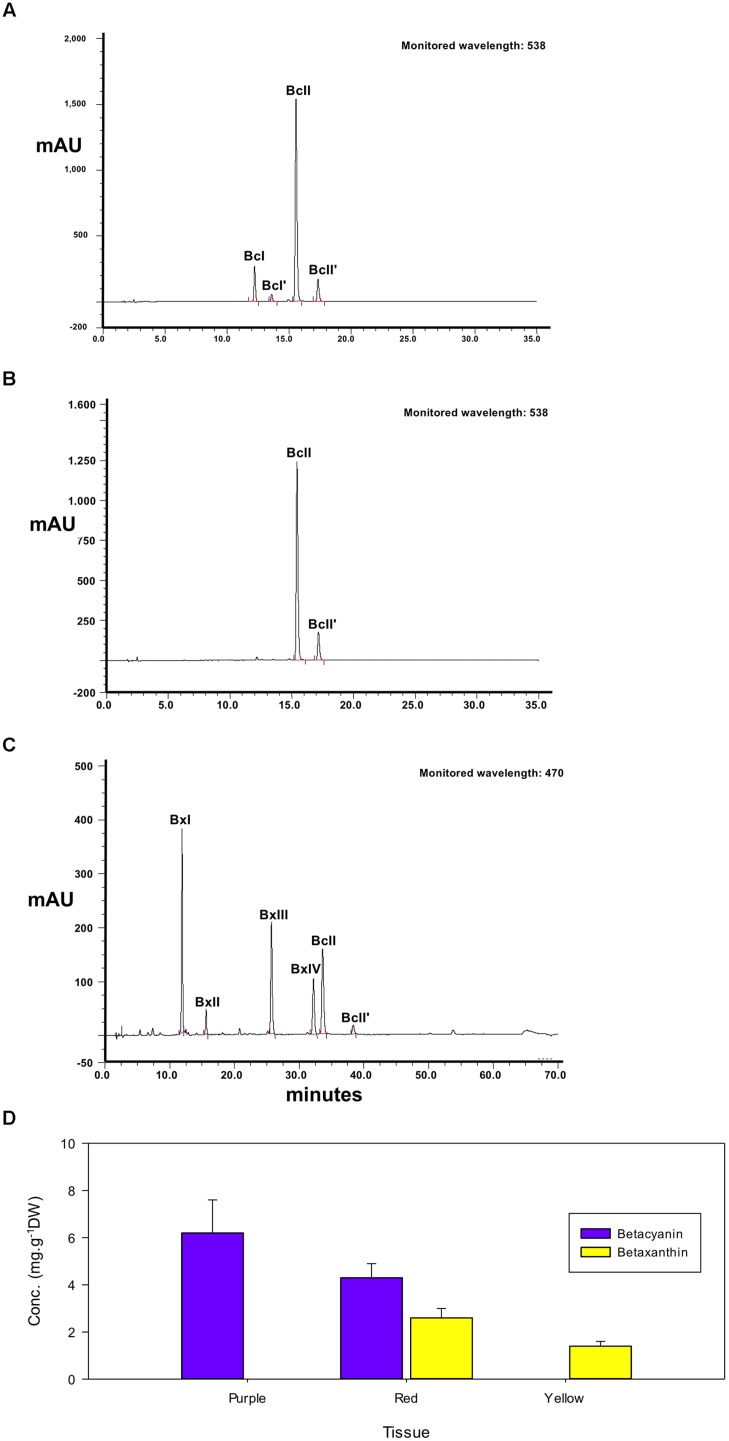
**High-performance liquid chromatography elution profiles of betalain pigment extracts from petals of *Parakeelya mirabilis*.** Extracts of violet **(A)** and red **(B)** petal sectors were separated by betacyanin elution gradient and monitored at 538 nm. Red **(C)** petal sectors were also separated by betaxanthin elution gradient and monitored at 470 nm. The calculated proportion of total betacyanins or betaxanthins in each petal sector is shown in **(D)**. BcI, betanin; BcI’, iso-betanin; BcII, betanidin; BcII’, iso-betanidin; BxI, vulgaxanthin I; BxII, vulgaxanthin II; BxIII, dopaxanthin; BxIV, portulacaxanthin II. The peak IDs are identical to those in **Table 1**.

Fully open flowers of *Parakeelya mirabilis* show four distinct color sectors of violet, white, red, and yellow (**Figure 2**). These were dissected and analyzed for betalain content (**Figure 2** and **3**), along with stamens, stigmata, ovaries, and leaves (**Figure 2**). It was not possible to avoid sampling a small amount of colored tissue when sampling the white sector tissue. The highest betalain content was detected in violet and red petal sectors followed by stigmata, yellow petal sectors and stamens. As was expected from the tissue dissection, a small amount of pigment was also detected in white petal sectors. Total betalain content increased markedly during petal development, with a major increase occurring between stages 2 and 3, when petals are still enclosed within the sepals. A low amount of betaxanthins was present from stage 1, while betacyanins were first detected at stage 2. Ovaries and leaves, which are green tissues, had relatively low betaxanthin content and contained no detected betacyanins.

High-performance liquid chromatography profiles showed that the violet petal sectors of *P. mirabilis* contained four betacyanins, with no betaxanthin apparent (**Figure 3**). Peaks at 12.2, 13.6, 15.5, and 17.3 min retention times in the betacyanin gradient represented the glycosides betanin and iso-betanin, and the aglycones betanidin and iso-betanidin, respectively, as identified by LC–MS (**Table 1**). The aglycone betacyanin accounted for the large majority of the total betacyanin. The yellow petal sectors contained four different betaxanthins, the major one being vulgaxanthin I followed by dopaxanthin, vulgaxanthin II, and portulacaxanthin II. Red petal sectors contained the same betaxanthins in a similar proportion and only the two betacyanin aglycones, betanidin, and iso-betanidin (**Figure 3**). Due to the wide absorption region of betacyanins, they can also be discerned in the betaxanthin HPLC separation monitored at 470 nm, wherein they contribute ∼30% of the total absorbance value at this wavelength (data not shown).

**Table 1:** Betalain pigment composition^a^ in *Parakeelya mirabilis* petals.

Peak^b^	Trivial name		Violet	Red	Yellow	R*t*^e^ (min)	λmax^f^ (nm)	m/z [M+H]^+^	m/z MS^2^
**Betacyanin^c^**		**Side chain**							
BcI	Betanin	5-*O*-glucoside	9.0	-	-	12.2	534	551	389
BcI’	Iso-betanin	5-*O*-glucoside	3.3	-	-	13.6	533	551	389
BcII	Betanidin	Aglycone	68.7	77.0	-	15.5	540	389	343
BcII’	Iso-betanidin	Aglycone	19.0	23.0	-	17.3	540	389	343
									
**Betaxanthin^d^**		**Amino acid**							
BxI	Vulgaxanthin I	Glutamine	-	56.0	56.0	11.9	469	340	323
BxII	Vulgaxanthin II	Glutamic acid	-	5.0	7.0	15.6	469	341	297
BxIII	Dopaxanthin	Dopa	-	25.0	30.0	25.7	470	391	347
BxIV	Portulacaxanthin II	Tyrosine	-	14.0	7.0	32.2	471	375	331

*^a^Proportion (%) of betacyanin and betaxanthin profiles as determined by peak integration.*

*-, not detectable.*

*^b^The peak IDs are identical to **Figure 3**.*

*^c^Separation in Betacyanin gradient monitored at 538 nm.*

*^d^Separation in Betaxanthin gradient monitored at 470 nm.*

*^e^Retention time.*

*^f^Wavelength of maximum absorbance.*

### Cloning and Expression Patterns of *Parakeelya* DOD and DOD-Like Sequences

To examine the genetic control of betalain biosynthesis in *P. mirabilis*, PCR was used to clone candidate LigB cDNAs from petal RNA. Using degenerate primers designed to Class II LigB sequences from other betalain species, cDNA fragments representing two different *LigB* genes were isolated. After RACE-PCR, full-length CDS was obtained for each. pBlast analysis using the NCBI non-redundant protein sequences database revealed that one showed highest identity to *Portulaca grandiflora DODA* (*PgDODA*; Q7XA48.1). As *PgDODA* has been shown to have a role in betalain production (Christinet et al., 2004) this gene was named *DOD*. *PmDOD* is 813 bp in length and encodes 271 amino acids. The other cDNA sequence had highest identity to *Phytolacca americana DOD1* (*PaDOD1*; BAH66635.1). The contribution, if any, of *PaDOD1* to betalain synthesis has not been resolved (Takahashi et al., 2009) therefore this *P. mirabilis* sequence was named *DOD-like*. *PmDOD-like* is 810 bp in length and encodes 270 amino acids. The *P. mirabilis* DOD and DOD-like amino acid sequences showed less identity to each other than to the heterologous sequences.

Spatio-temporal expression patterns of *DOD* and *DOD-like* in relation to betalain accumulation patterns in *P. mirabilis* were examined using quantitative PCR RNA analysis (**Figure 2**). During flower development, *DOD* transcript abundance correlated with the accumulation of betalain pigment while transcript abundance for *DOD-like* did not, and remained low. Transcript abundance was then compared across the different colored sectors within the petal as well as across other floral organs and leaves. Again, transcript abundance of *DOD*, but not *DOD-like*, generally correlated with betalain content (**Figure 2**). *DOD-like* transcript amounts were relatively low in all tissue samples. *DOD* was detected in the white sector at amounts similar to that for the violet and red sectors. This result was not an artifact of the sampling; the amount of contaminating pigmented tissue was only minor as indicated by the low amount of betalains detected (**Figure 2**), and DOPA feeding of the petal induced pigmentation in the white sector, indicating the presence of DOD activity (**Figure 2**). Overall, the results support a role for only *DOD* in betalain production, with *DOD-like* having a different and as yet unidentified role.

### *In planta* Assays of PmDOD and PmDOD-like Activities

**FIGURE 4:**
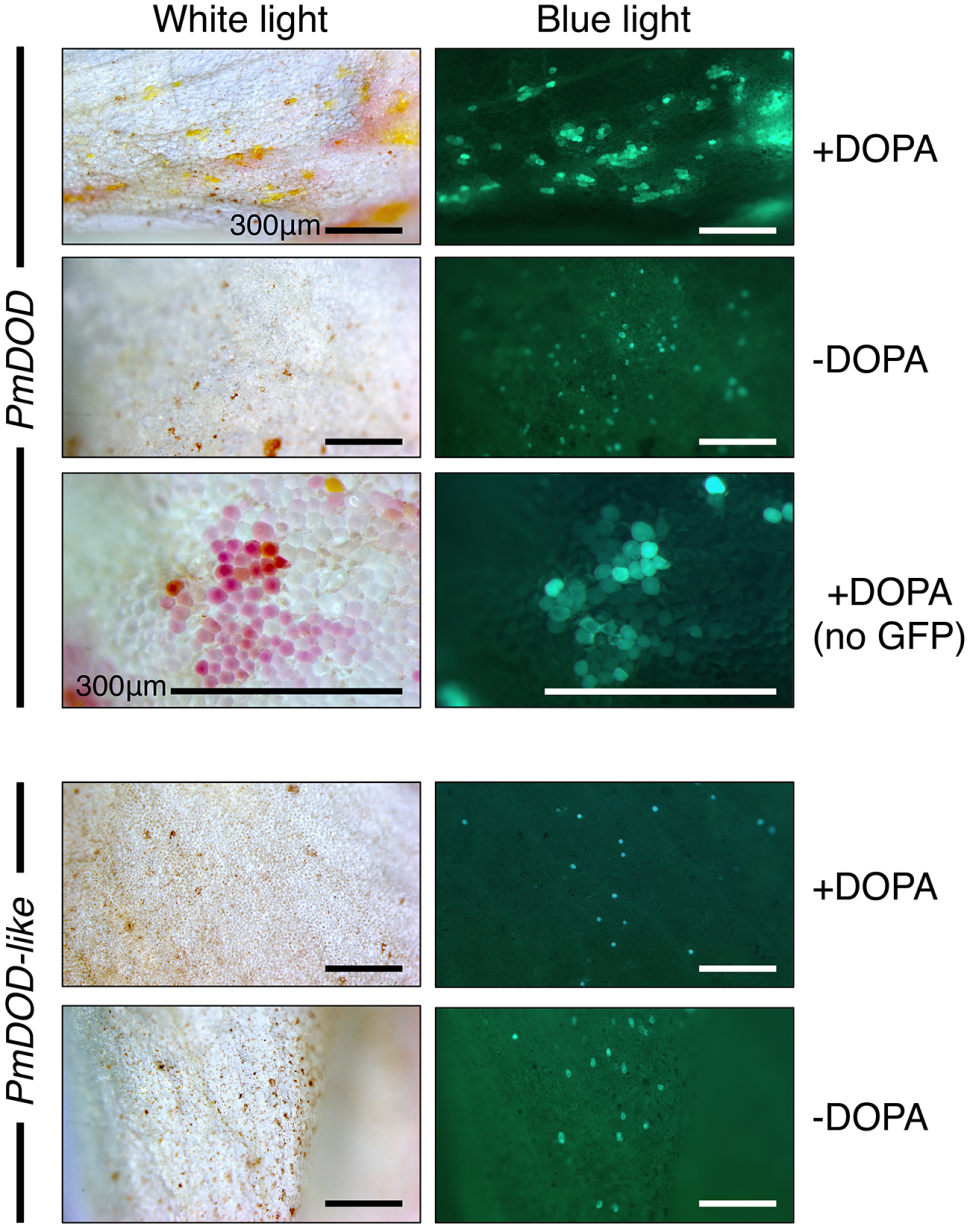
**Differential activity of *PmDOD* and *PmDOD-like* in a transient expression assay.** White petal tissue of *A. majus* was bombarded with constructs for the genes (driven by the *35S* promoter) followed by supplementation with DOPA or water. Representative petal samples are shown. A *GFP* construct was also included, except for the *PmDOD*-bombarded tissue in the magnified panels. Betalain pigments were observed only in *PmDOD*-bombarded tissue that was also fed DOPA and they had characteristic autofluorescence in blue light. No pigments were observed in *PmDOD-like* bombarded tissue despite GFP fluorescence indicating that the experimental conditions were sufficient for transformation. Scale bar = 300 μm.

**FIGURE 5:**
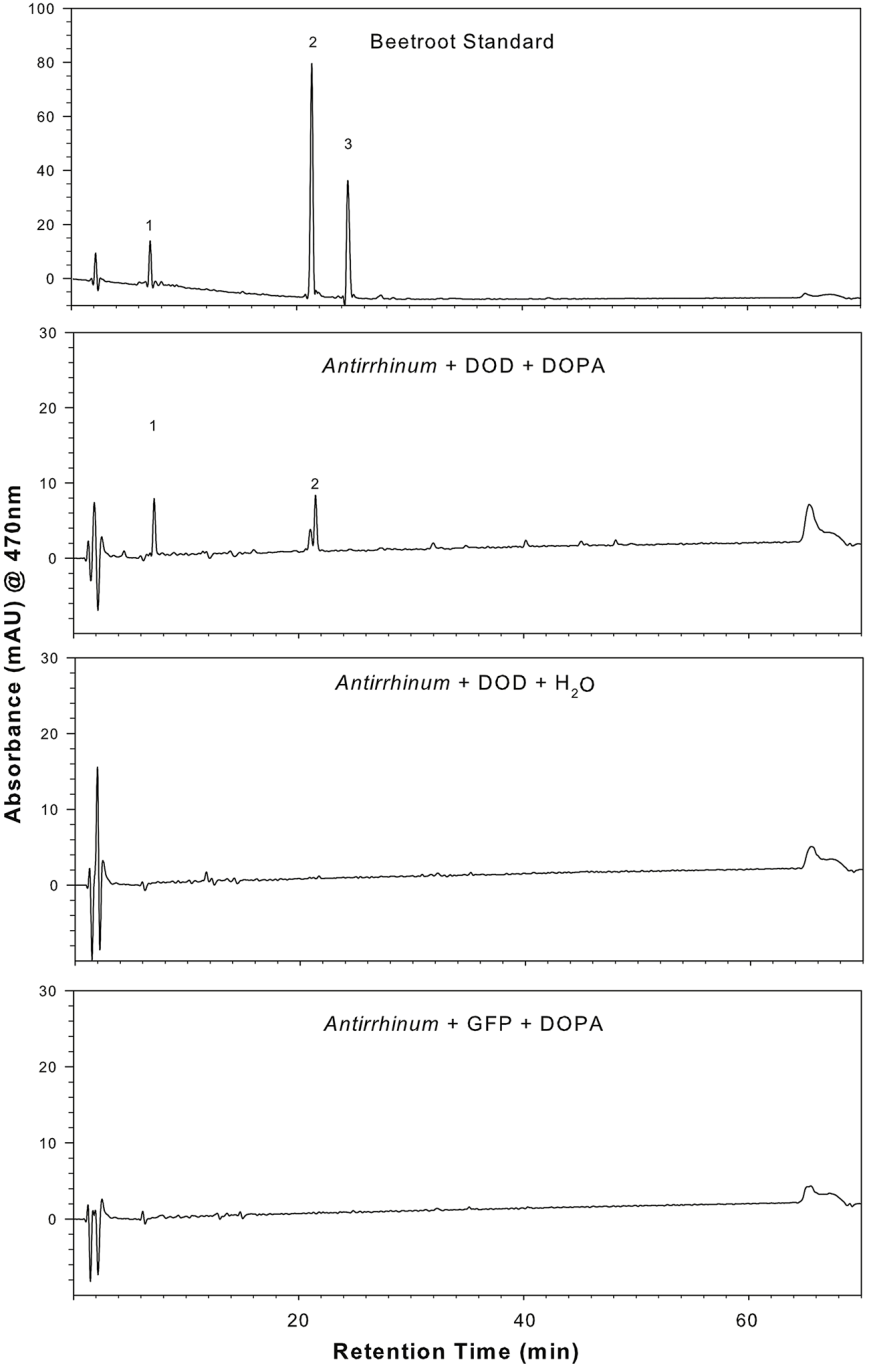
**Presence of betalain pigments in *A. majus* petals transiently transformed with *35S:PmDOD* and fed DOPA.** HPLC analysis was conducted on a tissue extract and compared to a known beetroot control sample. Pigment retention times and absorption maximums were consistent with the major pigments being the same as those in beetroot – betanin (peak 2) and vulgaxanthin I (peak 1). The chromatograms shown are only for the solvent gradient optimized for betaxanthin separation and detection at 470 nm, conditions that are also adequate for separation and detection of the beetroot betacyanins. Chromatograms of *A. majus* control tissue (*35S:PmDOD*-bombarded tissue fed water, *35S:GFP* bombarded tissue fed DOPA) show no pigment peaks.

The potential for DOPA 4,5-extradiol cleavage activity by DOD and DOD-like was assayed using a biolistics-based transient gene expression assay (Harris et al., 2012). This method uses the formation of betalain pigments as an indicator of 4,5-DOPA cleavage and betalamic acid (BA) formation. Petals of the white-flowered *nivea* (*chalcone synthase*) mutant of the anthocyanin-producing species *A. majus*, which lack anthocyanins and other flavonoids, were bombarded with the *P*. *mirabilis* cDNA constructs driven by the *35S* promoter and fed DOPA. A construct with a *Green Fluorescent Protein* (*GFP*) reporter gene (*35S:GFP*) was co-precipitated with the other constructs onto the gold particles in some experiments as an indicator of transformation frequency. Pigment production was observed only in tissue bombarded with *35S:DOD* and not in tissue bombarded with *35S:DOD-like* or in control tissue (bombarded tissue fed water; **Figure 4**). The pigmented zones showed green autofluorescence under blue light in the absence of GFP, a characteristic typical of betaxanthins (Gandía-Herrero et al., 2005). The results for *35S:DOD* were typical of what was observed in (Harris et al., 2012) when using a construct for a *Portulaca DOD* gene confirmed to be betalain-related. The pigments produced in the bombarded tissue were identified as the betacyanin betanin and the glutamine-based betaxanthin vulgaxanthin I based on HPLC comparison to the pigments in a beetroot standard sample (**Figure 5**). The pigment accumulated in multicellular zones, likely due to DOD protein or BA/pigment moving from the single transformed cells into adjacent untransformed cells. No pigment was observed in tissue bombarded with *35S:DOD-like* despite GFP fluorescence showing the transformation process was successful (blue light panels, **Figure 4**). The *DOD-like* cDNA used in these assays was from a different accession of *P*. *mirabilis* and has two amino acid changes (ORF E192Q and T265S, both outside the proposed catalytic domain) compared to the original *DOD-like* sequence, which may represent allelic variation. It was confirmed that these amino acid changes were not influencing the assay by repeating the experiment with a construct using the original *DOD-like* sequence, with the same results obtained (Supplementary Figure S1).

**FIGURE 6:**
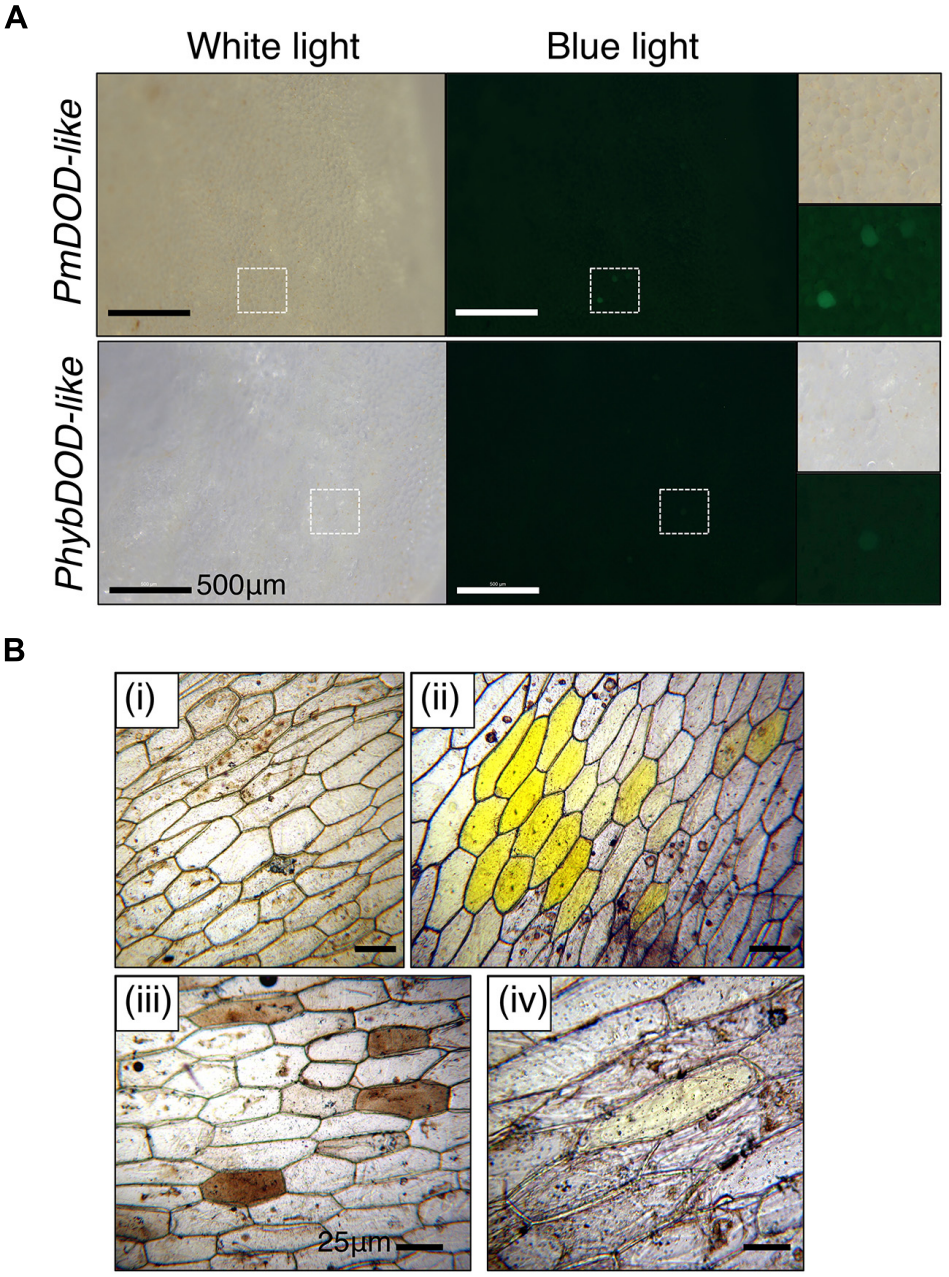
**Cell phenotypes in tissue bombarded with *DOD-like* constructs and fed DOPA.** Tissue bombarded with constructs for PmDOD-like and PhybDOD-like. The *GFP* construct was not included. **(A)** *A. majus* petal tissue used. Single green fluorescent cells at low frequency were observed under blue light. A few of these cells were possibly pale pink under white light in the PmDOD-like bombarded sample. Hatched squares show regions depicted at higher magnification in the panels on the right side. Scale bar is the same size in all. **(B)** White onion scale tissue used. No pigments observed when bombarded with gold particles only (i); conspicuous multicellular yellow zones observed when bombarded with the PmDOD construct (positive control; ii); brown cells (iii) and a rare pale yellow cell (iv) was observed when bombarded with the PhybDOD-like construct. Scale bar = 500 μm **(A)**, 25 μm **(B)**.

When the transient assay was repeated for *DOD-like* without including the GFP internal control, a phenotype was revealed under blue-light. Single cells with weak green autofluorescence were observed at low frequency (**Figure 6A**). These cells were generally not visibly pigmented under white light, except for a few that possibly had a very pale pink color, suggesting extremely limited betalain production. No cells with these phenotypes were observed in any control tissue; viz. bombarded tissue fed water or tissue bombarded with empty vector or the GFP reporter construct and fed DOPA (data not shown). In an attempt to enhance any weak phenotypes, the GFP-free experiment was repeated using the higher concentration of 10 mM DOPA (rather than 1 mM). Close examination of the sporadic blue light fluorescent foci revealed that some foci had more white-light visible pigmentation than had been observed in the previous experiment (Supplementary Figure S1), albeit it was still very weak. Compared to *DOD*, which gave abundant and multicellular betalain-pigmented foci that were readily apparent even under much lower magnification (**Figure 4**, Supplementary Figure S2), both *DOD-like* constructs gave very limited activity. The low frequency of the cells precluded biochemical analysis of the phenotype from *DOD-like*, but these results suggest that DOD-like has, at best, only a very limited capacity for a DOPA cleavage that would allow BA formation.

### The LigB Gene Family in *Parakeelya* and Other Caryophyllales

**FIGURE 7:**
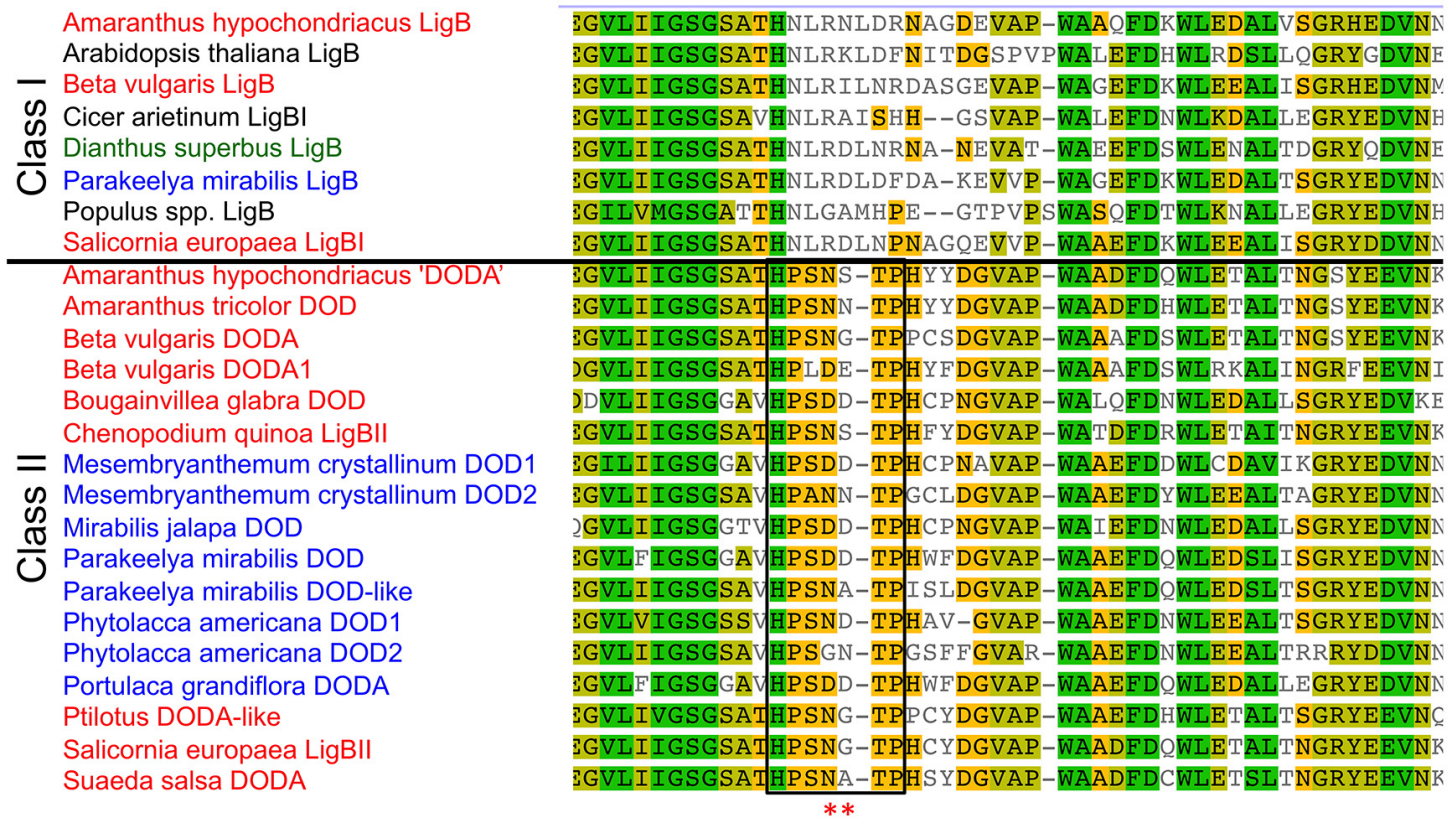
**Sequence alignment of catalytic domain and adjacent regions of plant LigB homologs.** The sequences are shown divided into Classes I and II. The catalytic domain is boxed and the asterisks mark the acidic residues that may be important for DOD activity/DOPA recognition. For the Class II genes that have DOD in their name (aside from this study) the gene names used are those in the GenBank annotations, which have generally been based only on sequence homology to DOD. The Amaranthaceae species names are in red, species of other betalain producing Caryophyllales families in blue, an anthocyanin taxa within the Caryophyllales (*Dianthus*) in green, and the non-Caryophyllales species in black. Accession numbers are given in Supplementary Figure S4.

Given the differences in gene expression and protein function of the *DOD* and *DOD-like* cDNAs, the LigB gene family was examined in more detail for *P*. *mirabilis* and other Caryophyllales species. Two transcriptome databases for *P*. *mirabilis*, formed using 454 Next Generation Sequencing for RNA from either the violet (22,665 transcript contigs) or yellow (21,189 contigs) regions of the petal, were interrogated for sequences with similarity to LigB sequences. In addition to the previously identified *DOD* and *DOD-like* sequences, a third candidate LigB sequence was identified (named *PmLigB*) that contained the H-N-L-R motif characteristic of Class I LigB proteins (Christinet et al., 2004), **Figure 7**, and Supplementary Figure S3). This had 59.6 and 67.4% deduced amino acid identity to *PmDOD* and *PmDOD-like*, respectively. Given the sequence divergence this suggests that there are at least three functionally distinct *LigB* genes in *P*. *mirabilis*.

Publically available sequence databases were interrogated to examine the occurrence of similar LigB gene families in non-Caryophyllales species and in betalain-producing or anthocyanin-producing species of the Caryophyllales. Examination of sequences from non-Caryophyllales species in NCBI/GenBank (http://www.ncbi.nim.nih.gov) found only Class I type sequences, as based on the conserved region (**Figure 7** and data not shown). Examination of sequences from anthocyanin-producing species of the Caryophyllales in GenBank and a transcriptome database for the Caryophyllaceae species *Silene vulgaris* (Sloan et al., 2012), also found only Class I type sequences (*Dianthus superbus* JL390264; *Silene vulgaris* isotig 21529). However, for the five betalain-producing families (11 species) of the Caryophyllales for which LigB sequences were available on NCBI, additional sequences were found that were more similar to *DOD* and *DOD-like* of *P*. *mirabilis* than they were to the Class I LigB (**Figure 7**). Including *P*. *mirabilis*, these represent the plant families of Amaranthaceae, Aizoaceae, Nyctaginaceae, Phytolaccaceae, and Portulacaceae.

**FIGURE 8:**
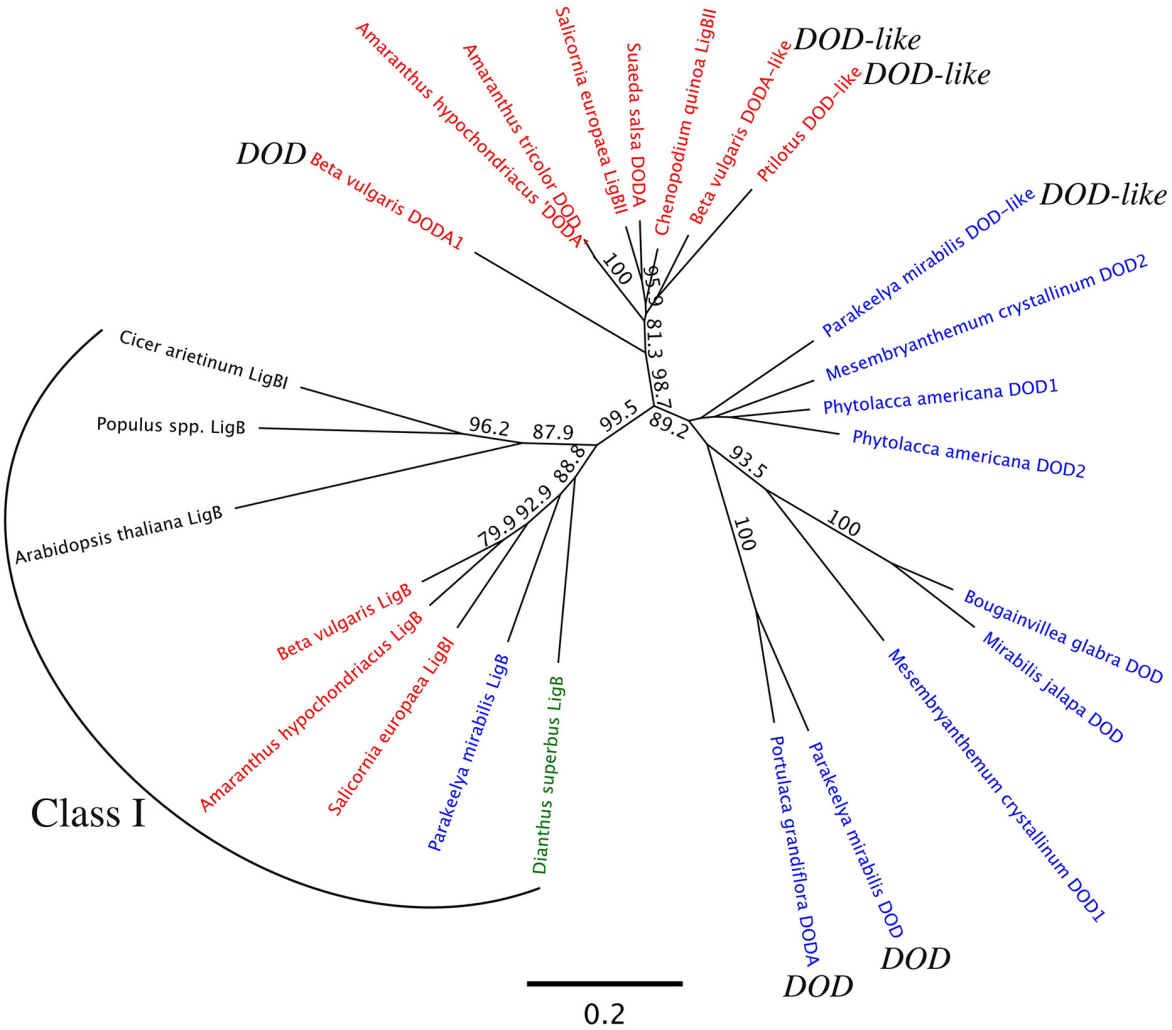
**Maximum likelihood analysis of LigB genes.** Analysis used the nucleotide sequences of the putative ORFs. The Amaranthaceae species names are in red, species of other betalain producing Caryophyllales families in blue, an anthocyanin taxa within the Caryophyllales (*Dianthus*) in green, and the non-Caryophyllales species in black. Sequences for McDOD1 and McDOD2 are not full-length but span the catalytic domain. Bootstrap analysis used 1000 datasets, with results shown for nodes that have at least 70% support. The branch-lengths indicate the average number of substitutions per site, with the scale bar given at the bottom of the figure. See Supplementary Figure S4 for the accession numbers. The Class I grouping is indicated. For the Class II sequences, the published DOD of *Portulaca grandiflora* is indicated along with the activities of sequences assayed in this study.

The relationships within the LigB family were examined by forming phylogenetic trees on the DNA sequences (**Figure 8**). The resultant tree shows a clear separation of Class I *LigB* sequences away from the additional LigB sequences found in betalain-producing species, with close to 100% bootstrap support. The additional *LigB* sequences form distinct clades consisting of those from the Amaranthaceae and those from the other betalain families examined, again with bootstrap support close to 100%. Within the non-Amaranthaceae clade, *PmDOD* and *PmDOD-like* are in separate groups with high bootstrap support, supporting the transient assay results that they have different biological activities. Within the Amaranthaceae species, the *Beta vulgaris DODA1* separates from *BvDODA*, with *BvDODA* being close to *PhybDOD-like* (of *Ptilotus*), suggesting they too may have distinct activities. Trees formed on the amino acid sequences, with or without outgroup rooting, gave a very similar outcome to that from the DNA sequence (Supplementary Figure S4).

### *In planta* Assays of the Function of Amaranthaceae *DOD* and *DOD-like* Sequences

**FIGURE 9:**
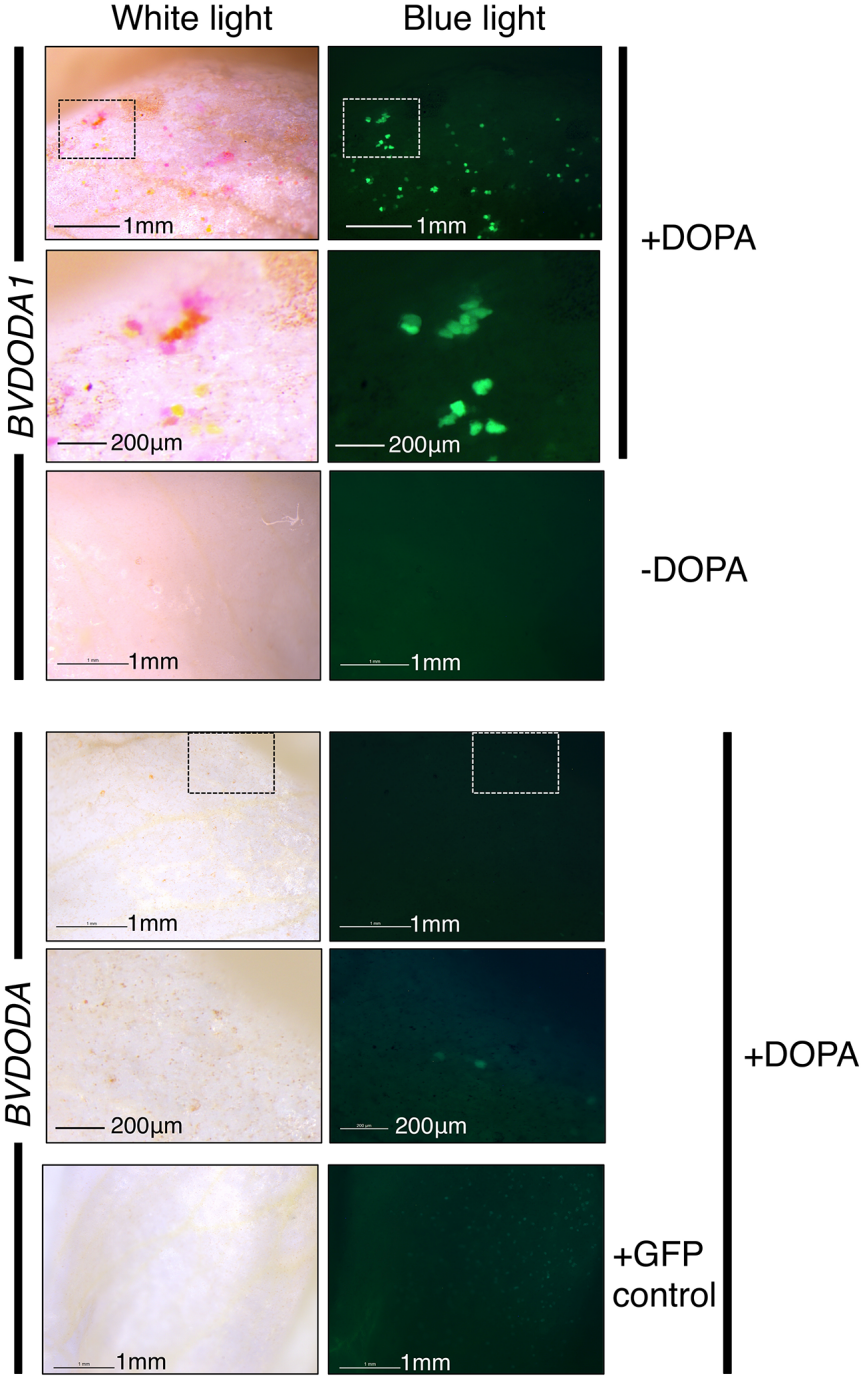
**Differential activity of *BvDODA* and *BvDODA1* in a transient expression assay.** White petal tissue of *A. majus* was bombarded with constructs for the genes (driven by the *35S* promoter) followed by supplementation with DOPA or water. Representative petal samples are shown. Pigmentation was only visible in *BvDODA1* bombarded tissue that was also fed DOPA. The pigmented regions also had fluorescence in blue light characteristic of betaxanthins. The only phenotype observed in *BvDODA*-bombarded tissue was a small number of cells in the DOPA-fed samples with weak green fluorescence in blue light. Control tissue bombarded with both *BvDODA* and *GFP* constructs and fed DOPA lacked pigment formation while GFP fluorescence indicated transformation had occurred. Scale bar = 1 mm. Hashed squares show regions depicted at higher magnification in panels with 200 μm scale bars.

Given the differential biological activities and phylogenetic separation of *PmDOD* and *PmDOD-like*, the biological activity of Amaranthaceae genes with sequence similarity to *DOD* or *DOD-like* was examined. The characterized sequences from *B. vulgaris* (*BvDODA* and *BvDODA1*) were used along with a Class II homolog isolated from green leaf tissue of *Ptilotus* (*PhybDOD-like*), another Amaranthaceae species. Based on the amino acid sequences, BvDODA is closer to PhybDOD-like (80.2% identity) than it is to BvDODA1 (70.1% identity). Outside of the Amaranthaceae (**Table 2**), BvDODA is also more similar to PmDOD-like than PmDOD (or BvDODA1). The differences in % identities when examining BvDODA1 and PmDOD are less conclusive (**Table 2**), but in general they suggest that BvDODA, PhybDOD-like, and PmDOD-like may have a divergent enzymatic activity to that of BvDODA1 and PmDOD. Previous analysis has suggested BvDODA1 is the key LigB gene for betalain biosynthesis in *B. vulgaris* (Hatlestad et al., 2012), although BvDODA is also expressed in colored tissues (Hatlestad et al., 2012) and recombinant protein has some DOPA 4,5-cleavage activity (Sasaki et al., 2009; Gandía-Herrero and García-Carmona, 2012). The transient gene expression assay was used with constructs driven by the *35S* promoter. Pigment production upon supplementation with DOPA was observed only in *35S:BvDODA1*-bombarded tissue and not when *35S:BvDODA* or *35S:PhybDOD-like* was used (**Figure 9**). However, as seen with *PmDOD-like* (**Figure 4**), tissue bombarded with these other constructs had a low frequency of single cells with pale green autofluorescence under blue-light, but no visible pigmentation under white light (**Figure 9**). The transient expression assay was repeated for *PhybDOD-like* using *Allium cepa* (onion) epidermal cells, with *PmDOD* as a positive control. After DOPA feeding, yellow multicellular zones developed in *PmDOD* bombarded tissue, while sporadic brown cells and a single pale yellow cell developed in tissue bombarded with *PhybDOD-like* (**Figure 6B**). This demonstrated that the differential activity was not contingent on the host tissue used in the assay. These results provide further evidence of Class II LigB proteins in betalain producing taxa that are functionally different to DOD. These DOD-like enzymes, however, may conditionally exhibit limited DOPA cleavage activity that leads to BA.

**Table 2:** Amino acid identify values (%) for deduced DOD and DOD-like sequences from *Beta vulgaris* and *Parakeelya mirabilis*, and DOD-like from *Ptilotus* sp. (Phyb), based on pairwise alignment.

	BvDODA1	BvDODA(-like)	PmDOD	PmDOD-like	PhybDOD-like
BvDODA1		70.1	57.4	64.9	66.4
BvDODA(-like)	70.1		60.7	73.9	80.2
PmDOD	57.4	60.7		70.4	60.4
PmDOD-like	64.9	73.9	70.4		70.5
PhybDOD-like	66.4	80.2	60.4	70.5	

A Class I LigB Caryophyllales representative (from *Dianthus superbus*, carnation) was also tested in the transient expression assay. No visible pigmentation or fluorescence under blue light was observed (Supplementary Figure S2), indicating that this class of protein does not have the same activity on DOPA in this assay as Class II types.

### The Genomic Structure of the LigB Family in *Beta vulgaris*

**FIGURE 10:**
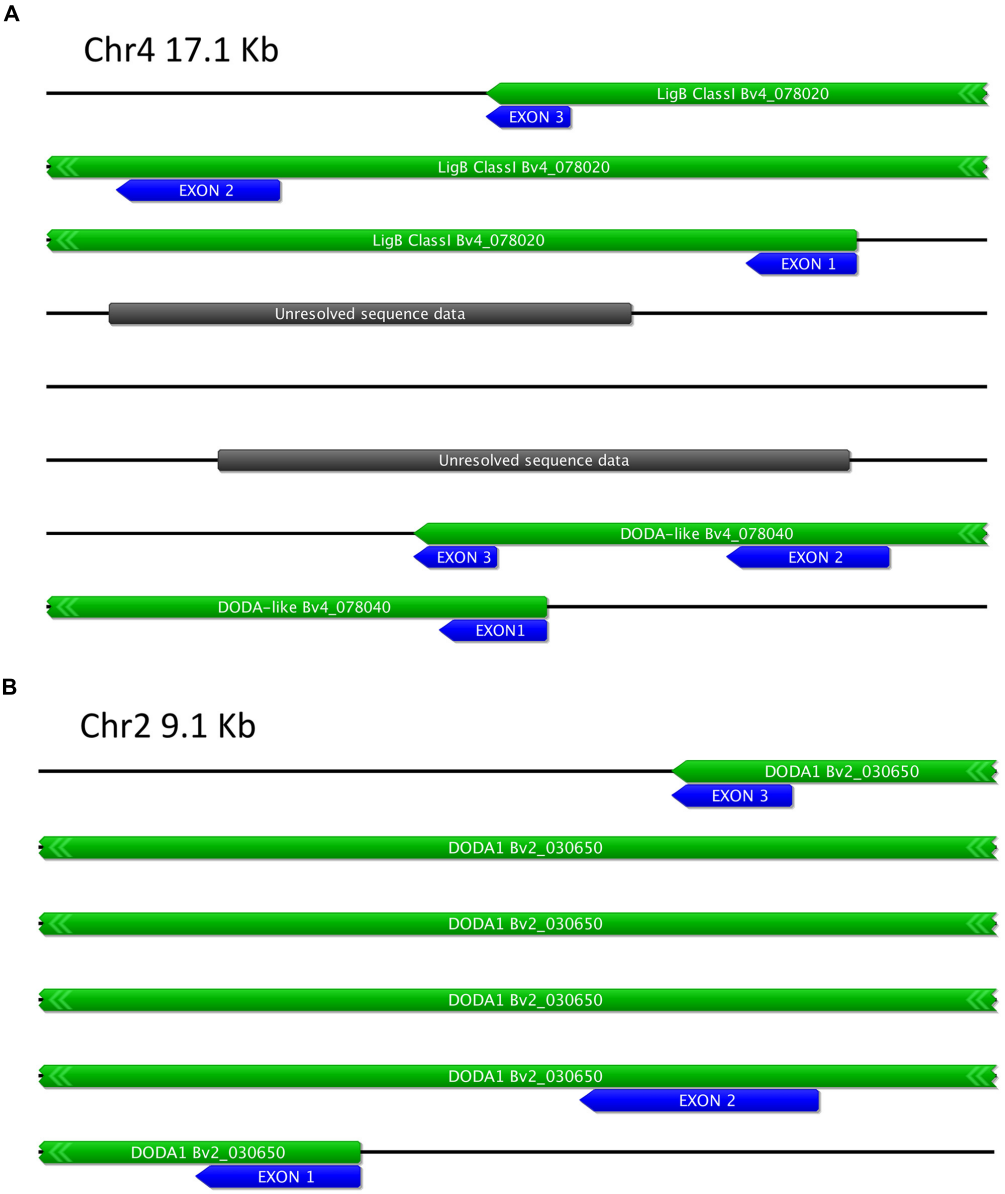
**Schematic of *Beta vulgaris* LigB genomic regions. (A)** Region of Chr4 containing two related LigB genes, termed *LigB Class I* and *DODA-like* [BvDODA HQ656022]. **(B)** Region of Chr2 containing *DODA1* [HQ656027]. The predicted transcript regions are marked with green boxes and the exons with blue boxes. Additional gene labeling is as per the Augustus annotation of the genome at the *Beta vulgaris* Resource (Dohm et al., 2014).

Diversification of gene function in plant secondary metabolism during evolution often involves localized gene duplication followed by neofunctionalisation, resulting in the tandem occurrence of related biosynthetic genes (Ober, 2005). The only betalain-producing species for which a genome sequence is publically available is *B. vulgaris* (Dohm et al., 2014), so the structure and localisation in the genome of the three LigB sequences examined here was determined (**Figure 10**). All three genes have three exons, and the exon size and position of introns is conserved, with exon 1 being between 246 to 260 bp, exon 2 371 to 378 bp and exon 3 190 to 193 bp (**Figure 10**). *BvDODA1* is located on Chr2 while both the *ClassI LigB* and *DODA* are located on Chr4. The two genes on Chr4 are on the same genomic sequence scaffold, with the end of exon 3 of *DODA* being ∼7.6 Kb upstream of exon 1 of the *ClassI LigB* gene (**Figure 10**).

### Characterization of the Catalytic Domain of DOD and DOD-like

The residues in the motif underlying our classification of the *LigB* genes are postulated to have a role in substrate recognition (Christinet et al., 2004). Given the differential activities in the transient assay it was examined whether there were conserved residues in the catalytic domain that may define a DOD protein (regardless of family taxon). Alignment of the sequences shows two adjacent acidic residues (D-D/E) in all confirmed DOD sequences that are also present in all sequences that are predicted to be DOD based on the phylogenetic analysis (**Figure 8**). To determine whether these residues are the reason of the differential activity observed in the transient assays, the predicted catalytic domain of PmDOD-like was mutagenised to convert HPSNATP to HPSDDTP, and the construct tested in the transient assay. The altered catalytic domain did not give activity that was characteristic of PmDOD (Supplementary Figure S2). However compared to the results with PmDOD-like, there were pigmented foci that were more readily detectable due to higher levels of pigmentation (Supplementary Figure S2).

## Discussion

To gain further understanding of the betalain biosynthetic pathway, we have examined the pathway in *Parakeelya mirabilis* of the Montiaceae, which is phylogenetically well separated within the Caryophyllales from the established model of *Beta vulgaris*. Yellow betaxanthins were found at low amounts in leaves and ovaries and at higher amounts in all of the other floral organs examined. The red/purple betacyanins were only found at significant amounts in the petals, with only trace amounts present in the stamens and stigmata. The petals contain both betacyanins and betaxanthins, with betacyanin aglycones (betanidin and iso-betanidin) accounting for the large majority of the total betacyanin in the petals. Betacyanins, like anthocyanins, are typically glycosylated compounds. The role of glycosylation for betacyanins has not been established, but for anthocyanins it is essential for pigment stability and localisation into the vacuole (Tanaka et al., 2008; Cheynier et al., 2013). Although the accumulation of aglycone betacyanin is highly unusual, it has been previously reported in some species of the Portulacaceae and Aizoaceae, and in the Aizoaceae its presence was associated with violet flower colors while betanidin 5-*O*-glucoside (betanin) was associated with red flower colors (Strack et al., 1990). In *P. mirabilis* the area of betacyanins alone is also violet in color, while the red color is generated by the additional presence of yellow betaxanthins. Betacyanin aglycone occurrence in *P. mirabilis* is unlikely to be due to a lack of an appropriate glycosyltransferase gene, as some betanin and iso-betanin (which are glycosylated) was present.

The coloration of the *P. mirabilis* petals consists of violet, red, and yellow zones, with the violet and red separated by a colorless (white) zone. The violet petal sectors contained four betacyanins, with no betaxanthin apparent, while the yellow petal sectors contained four different betaxanthins, with no betacyanins apparent. The red sector color was the result of a mix of both betacyanins and betaxanthins. Also, the betaxanthins were present in the smallest flower buds examined, while betacyanins only appeared later in flower development. Thus, the flower has a series of distinct regulatory zones for betalain biosynthesis in which betacyanin and betaxanthin biosynthesis is separated both spatially and temporally. There has been no research on how such zones of betalain biosynthesis may be regulated at the molecular level. However, the formation of anthocyanin-based flower coloration patterns has been the focus of much research and R2R3MYB genes shown to be key to establishing such patterning in a range of species (Shang et al., 2011; Davies et al., 2012; Albert et al., 2014; Yamagishi et al., 2014; Yuan et al., 2014). As the only regulatory gene identified to date for betalain biosynthesis is an R2R3MYB that has proposed to be co-opted from anthocyanin regulation (Hatlestad et al., 2015), such genes would be excellent candidates to examine for the basis of the flower color patterning in *P. mirabilis* petals.

A cDNA for a protein with Dopa 4,5-dioxygenase (DOD) activity was identified from *P. mirabilis* and its transcript abundance examined across the same samples for which betalain analysis was conducted. The temporal pattern of DOD transcript during petal development closely reflected the pattern of betalain accumulation and also correlated to betalain amounts in the leaves, ovaries, stigmata and stamens. There are relatively few studies on whether transcript abundance for proteins with proven DOD activity correlates with betalain production. The clearest example is that *BvDODA1* transcript is found at high amounts in betalain producing tissues of *B. vulgaris* (Hatlestad et al., 2012), and the *BvDODA1* promoter is activated by the R2R3MYB betalain pathway activator (Hatlestad et al., 2015). The *P. mirabilis* data suggests regulation of the DOD gene expression is also a key aspect of regulation of the betalain biosynthetic pathway in this species. However, although the DOD transcript amounts correlated well with the overall amounts of betalains in *P. mirabilis*, the only variation across the different colored sectors of the petal was that amounts were lower in the yellow sector. The DOD transcript amounts in the white sector were similar to that in the red and violet sectors, and DOPA feeding resulted in pigmentation, suggesting that the spatial regulation of betacyanin and betaxanthin production in these sectors operates at a different step than through transcriptional regulation of the DOD gene.

In addition to a cDNA for DOD, a second cDNA encoding a DOD-like protein was identified from *P. mirabilis*. The *DOD-like* transcript abundance did not correlate to betalain levels, either in different tissues or across flower development (**Figure 2**), suggesting it has a role unrelated to betalain biosynthesis. Examination of public databases identified sequences with high % identity to either *DOD* or *DOD-like* from a range of Caryophyllales species. These ‘Class II’ LigB sequences showed clear separation from the ‘Class I’ type on amino acid alignments (**Figure 7**) and phylogenetic trees formed from either the nucleotide or deduced amino acid sequences (**Figure 8** and Supplementary Figure S4). Within the Class II, the Amaranthaceae species separated from representatives from four other Caryophyllales families. In the non-Amaranthaceae grouping of these sequences there was a separation that was not on a species basis, with groupings containing the *DOD* and *DOD-like* sequences whose function was assayed in this study. There were insufficient numbers of sequences to determine if this was also the case for the Amaranthaceae species, but BvDODA1 did separate from BvDODA. Given this suggestion of a larger LigB gene family consistently occurring in betalain producing plants, and the transcript abundance of *PmDOD-like* not reflecting betalain occurrence in *Parakeelya*, the function of *PmDOD-like* and *DOD-like* sequences from two Amaranthaceae species (*B. vulgaris* and *Ptilotus*) was examined using the transient assay system. None of the three sequences conferred betalain production in the way that *DOD* sequences did (**Figures 4,6**, and **9**), further suggesting that the *DOD-like* ones encode a distinct enzyme activity. What the activity (or activities) might be is unknown, but the *DOD-like* transgenes did confer production of weakly fluorescent and pigmented cells. Thus, it may be an activity closely related to that of DOD. In this regard, it should be noted that while in our assay the DOD-like BvDODA did not demonstrate equivalent activity to confirmed DOD proteins, BA formation *in vitro* using recombinant BvDODA protein produced in *Escherichia coli* has been reported (Sasaki et al., 2009; Gandía-Herrero and García-Carmona, 2012) and there is a preliminary report of DOPA 4,5-cleavage activity *in vitro* for recombinant versions of some non-Caryophyllales plant *LigB* genes (presumably Class I types; Sasaki et al., 2007). However, it is difficult to know how reflective of *in planta* activity these *in vitro* assays are, as no comparative data has been presented for a DOD protein that has a confirmed role in betalain synthesis.

Although a distinct region of the peptide can distinguish Class I and Class II sequences, no single conserved motif was apparent that could be used to further separate sequences within Class II that might account for the different activities observed with DOD from DOD-like (**Figure 7** and Supplementary Figure S3). However, a comparison of the region postulated to be involved in substrate recognition (Christinet et al., 2004) revealed two adjacent acidic residues that may be important to DOD. These are found in PgDODA, PmDOD, and BvDODA1 (**Figure 7**), all of which gave clear betalain production in the transient expression assay (this paper and Harris et al., 2012). However, the lack of DOD-equivalent activity in the transient assay by PmDOD-like that had been mutated to gain these acidic residues found in DOD showed that on their own they were not sufficient to convert a DOD-like protein to DOD. Therefore, all the key residues that differentiate DOD from DOD-like are yet to be defined. Although the substituted residues did not generate DOD-equivalent activity, there was a visual increase in the weak pigmentation that could be produced in transformed cells, indicating that the residues may make some contribution to enzymatic efficiency and would be worthy of further investigation.

Published studies on the transcript abundance patterns for some of the DOD-like sequences from other species (as based on our alignments) also suggest they may not be involved in betalain biosynthesis and could be a part explanation of why ‘*DOD’* transcript levels often do not always correlate with betalain production. The stress induced *DODA1* and *DODA2* of *Amaranthus hypochondriacus* (Casique-Arroyo et al., 2014) likely encode *DODA-like* and *LigB* Class I sequences, respectively. The transcript abundance pattern of these genes did not consistently match the pattern of betalain production with regard to drought or herbivory stress, an observation that also led Casique-Arroyo et al. (2014) to suggest *AhDODA2* may have a role outside of betalain biosynthesis. Similarly, the stress induced *DODA* of *Suaeda salsa* is also likely a *DODA-like* sequence (Zhao et al., 2010, 2011), and its transcript abundance also only correlated with betalain production in some of the studies.

Only Class I *LigB* genes were found in the databases for taxa outside the Caryophyllales while both classes of *LigB* genes were found in a range of Caryophyllales species. One possibility is that a Class I gene duplication event gave rise to the Class II gene lineage, as this is a common event in plant secondary metabolism gene evolution (Ober, 2005). Such duplication often results in the tandem occurrence of related biosynthetic genes, and this was found to be the case when examining the *B. vulgaris* genome (the only betalain species for which a genome sequence is publically available). The conserved exon/intron structure of the three *B. vulgaris* genes examined here and the co-localization of the *Class I LigB* and *DOD-like* genes (**Figure 10**) supported a common gene ancestry. Investigation of the gene structure in other species would be of much interest to help answer the questions on the LigB gene family evolution.

## Author Contributions

H-HC, HN, BM, KC, and KS conducted experiments; KS, KD, and RC conducted sequence analyses; DL contributed to experimental analyses; KS, DH, KD, DJ contributed to project conception, experimental design, data interpretation; KG, KS, and KD supervised KC; DH and DJ supervised H-HC and BM; KS, DH, KD, and H-HC prepared the manuscript; All authors read and approved the final manuscript.

## Conflict of Interest Statement

The authors declare that the research was conducted in the absence of any commercial or financial relationships that could be construed as a potential conflict of interest.
