# Supplementary material for: Characterisation of betalain biosynthesis in Parakeelya flowers identifies the key biosynthetic gene DOD as belonging to an expanded LigB gene family that is conserved in betalain-producing species
Source: Front Plant Sci. 2015 Jul 7;6:499. doi: 10.3389/fpls.2015.00499 (PMC4493658; doi:10.3389/fpls.2015.00499)

***Supplementary Material***

**Characterisation of betalain biosynthesis in *Parakeelya* flowers identifies the key biosynthetic gene DOD as belonging to an expanded LigB gene family that is conserved in betalain‐producing species**

**Hsiao-Hang Chung^1,†^, Kathy E. Schwinn^2,*^, Hanh M. Ngo^2,^^, David H. Lewis^2^, Baxter Massey^1,§^, Kate E. Calcott^2,3,#^, Ross Crowhurst^4^, Daryl C. Joyce^1^, Kevin S. Gould^3^, Kevin M. Davies^2^, and Dion K. Harrison^1,‡^**

^1^The University of Queensland, School of Agriculture and Food Sciences, Centre for Native Floriculture, Gatton, QLD, Australia

^2^New Zealand Institute for Plant & Food Research Limited, Private Bag 11600, Palmerston North 4442, New Zealand

^3^Victoria University of Wellington, PO Box 600, Wellington 6140, New Zealand

^4^New Zealand Institute for Plant & Food Research Limited, Private Bag 92169, Auckland 1142, New Zealand

Current addresses: †Agricultural Biotechnology Research Center, Academia Sinica, Taipei, 11529 Taiwan; ‡InnoV8 Botanics, 12 Takara Court, Karana Downs, QLD 4306, Australia; §3/21 Barlow St, Scullin, ACT 2614, Australia; ^11646 Sunlit Leaf Ct, Houston, Texas, 77038, USA; #Ministry for Primary Industries, PO Box 2526, Wellington 6140, New Zealand

*** Correspondence:** Kathy E. Schwinn, New Zealand Institute for Plant & Food Research Limited, Private Bag 11600, Palmerston North 4442, New Zealand.

[Kathy.schwinn@plantandfood.co.nz](mailto:Kathy.schwinn@plantandfood.co.nz)

1. **Supplementary Figures and Tables**

**Supplementary Table 1: Sequences of DNA primers.**

| **Primer** | **Direction** | **Sequence (5**'**-3**'**)** |
| --- | --- | --- |
| **Degenerate oligonucleotides** | | |
| DOD F1 | For | ASYTTCTTYWTKTCTCATGGRAMTCCA |
| DOD R2 | Rev | TGGAAGTGAACTTGTARGAGSCRTA |
| EF1α F1 | For | GCYGAGATGMACAARMGGTCMTTC |
| EF1α R1 | Rev | CRATCTGDCCAGGGTGGTTCATG |
| ACT F1 | For | GCWGTSTTCCCMAGTATHGTTGGT |
| ACT R1 | Rev | TTDATRTCMCKBACAATTTCYCGYTC |
| **3' RACE** | | |
| 3'RT-primer |  | GACCACGCGTATCGATGTCGACTTTTTTTTTTTTTTTTV |
| DOD1 First PCR | For | TCACCACTTTAACGTGGGTCGT |
| DOD1 Semi-nested PCR | For | CCAAACAAAAGCACCATTAGGGTG |
| DOD2 First PCR | For | TTGGGAAAAGCATTGGCTCCTC |
| DOD2 Semi-nested PCR | For | ATCAAGCGAAAGCACCAAACTGG |
| Universal reverse primer | Rev | GACCACGCGTATCGATGTCGAC |
| **5' RACE** | | |
| RT-primer |  | Phosphate-GTGCAACGGGTAGA |
| DOD1 First PCR F | For | TCACCACTTTAACGTGGGTCGT |
| DOD1 First PCR R | Rev | CCCTATCGAACCCTGCTGACA |
| DOD1 Nested PCR F | For | CCAAACAAAAGCACCATTAGGGTG |
| DOD1 Nested PCR R | Rev | CTCGGGATTTTCTCCGGCAGA |
| DOD2 First PCR F | For | TTGGGAAAAGCATTGGCTCCTC |
| DOD2 First PCR R | Rev | GACCTCTCTTCTTGTCTACTACGCAT |
| DOD2 Nested PCR F | For | ATCAAGCGAAAGCACCAAACTGG |
| DOD2 Nested PCR R | Rev | GCTGGTACATAGGAGCAGGAAAGC |
| **Full-length amplification *PmDOD1 and 2* ^a^** | | |
| PmDOD1 F | For | AGCTAGGTACCATGGGTGTTGGGAGGAGGATG |
| PmDOD1 R | Rev | AGCTATAAGCTTTGGTAAACGAGACGACGAAC |
| PmDOD2 F | For | AGCTAGGTACCATGGGTGATCATGAAGCAATTC |
| PmDOD2 R | Rev | AGCTATAAGCTTGCCAATGACGACGAGTAC G |
| **Quantitative PCR primers and labelled probes** | | |
| DOD1 F | For | GGCTCGAGGATTCTCTCATTTC |
| DOD1 R | Rev | CCCTAATGGTGCTTTTGTTTGG |
| DOD1 Probe |  | 6FAM-CACATCTTCATACCTTCCA-MGBNFQ |
| DOD2 F | For | GATCAATGGCTAGAAGATAGTCTCACTAG |
| DOD2 R | Rev | AGTTTGGTGCTTTCGCTTGATAAT |
| DOD2 Probe |  | 6FAM-CACATCTTCATACCTTCCA-MGBNFQ |
| EF1α F | For | TGGGTACAACCCCGACAAA |
| EF1α R | Rev | GTTGTCTCCCTCAAATCCAGAGA |
| EF1α Probe |  | 6FAM-TCCCATTCGTTCCC-MGBNFQ |
| ACT F | For | GAGCAATTGGGATGACATGGA |
| ACT R | Rev | GCAACACGAAGCTCGTTGTAGA |
| ACTIN Probe |  | 6FAM-AAGATCTGGCATCACAC-MGBNFQ |

^a^ The underlined nucleotides mark the recognition sites for KpnI (GGTACC) and HindIII (AAGCTT).

## Supplementary Figure 1: Additional PmDOD-like transient activity assays. (A) A construct made with the original *PmDOD-like* sequence replicates results in Figure 4 i.e. it has differential activity to *PmDOD* in antirrhinum petals. Expression of GFP in the tissue bombarded with *PmDOD-like* and *35S:GFP* co-precipitated constructs demonstrated successful transformation. However betalain pigments were only observed in tissue bombarded with *PmDOD* and fed DOPA (1mM). (B) *PmDOD-like* gives sporadic foci that are pigmented and autofluorescent when bombarded into antirrhinum petals. Petals were fed 10mM DOPA, and the GFP construct was not included in this experiment. Arrows indicate a pigmented foci that is shown in higher magnification in the bottom panels. In (B), white and black balances were independently altered and colour saturation and contrast were increased to aid clarity. Constructs were made from synthesized ORFs.





**Supplementary Figure 2. (A) *DsLigB* (Class I) gives no phenotype when bombarded into antirrhinum petal tissue.** No phenotype was visible under white light, despite positive GFP signal demonstrating successful transformation. Also there was no phenotype under blue light (in sample bombarded without *35S:GFP* and in region**s** where gold particles were visible). The positive control tissue, bombarded with the *PmDOD* construct, had many pink and yellow multicellular foci showing autofluorescence under blue light, and these were readily visible under lower magnification. **(B) A mutagenized version of *PmDOD-like* does not have PmDOD-equivalent activity.** The catalytic domain of PmDOD-like was mutagenized to resemble that of PmDOD. This mutated version did result in some sporadic pigmented foci (indicated by arrows), but the result was substantially different to that obtained with *PmDOD* (see (A)). A *35S:GFP* control was included in this experiment. To aid clarity of the images in (B), white and black balances were independently altered and colour saturation and contrast were increased. All samples were fed 10mM DOPA.





**Supplementary Figure 3: LigB deduced amino acid sequence alignment.** Sequences were aligned by ClustalW followed by manual adjustment.

**Supplementary Figure 4: Phylogenetic analysis of LigB deduced amino acid sequences.** Analysis used the predicted ORF amino acid sequences. Sequences for McDOD1 and McDOD2 are not full-length but span the catalytic domain. The Amaranthaceae species names are in red, species of other betalain producing Caryophyllales families in blue, an anthocyanin taxa within the Caryophyllales (*Dianthus*) in green, and the non-Caryophyllales species in black. The Class I grouping is indicated. For the Class II sequences, the published DOD of *Portulaca* *grandiflora* is indicated along with the activities of sequences assayed in this study. The upper tree is formed with the *Populus* LigB sequence as the outgroup using MrBayes, with posterior probability values shown. The lower tree is formed without an outgroup using Maximum Likelihood and Bootstrap analysis of 1000 datasets, with results shown for nodes that have at least 70% support. The branch-lengths indicate the average number of amino acids substitutions per site, with the scale bar given at the bottom of the figure. See Supplementary Figure 3 for the sequence alignment used for the analyses. GenBank accession numbers: *Amaranthus* *hypochondriacus* LigB [AHV78224], *A.* *hypochondriacus* 'DODA' [ADZ48644], *Amaranthus* *tricolor* DOD [KP165399], *Arabidopsis* *thaliana* LigB [NM_117597], *Beta* *vulgaris* LigB [see Figure 9], *B.* *vulgaris* DODA1 [HQ656027], *B.* *vulgaris* DODA [AJ583017], *Bougainvillea* *glabra* DOD [AB435373], *Chenopodium* *quinoa* LigBII [CN781996], *Cicer* *arietinum* LigB [LOC101505085], *Dianthus* *superbus* LigB [JL390264], *Mesembryanthemum* *crystallinum* DOD1 [BF480453], *M.* *crystallinum* DOD2 [BE131205], *Mirabilis* *jalapa* DOD [AB435372], *Phytolacca* *americana* DOD1 [BAH66635], *P. americana* DOD2 [BAH66636], *Parakeelya* *mirabilis* DOD [KF747352], *P.* *mirabilis* DOD-like [KF747353], *Populus* *spp*. LigB [DT510620], *Portulaca* *grandiflora* DODA [AJ580598], *Ptilotus* *hyb*. DOD-like [KF747354], *Salicornia* *europaea* LigBI [GAIA01075260], *S.* *europaea* LigBII [GAIA01011644], *Suaeda* *salsa* DODA [ACO59903].

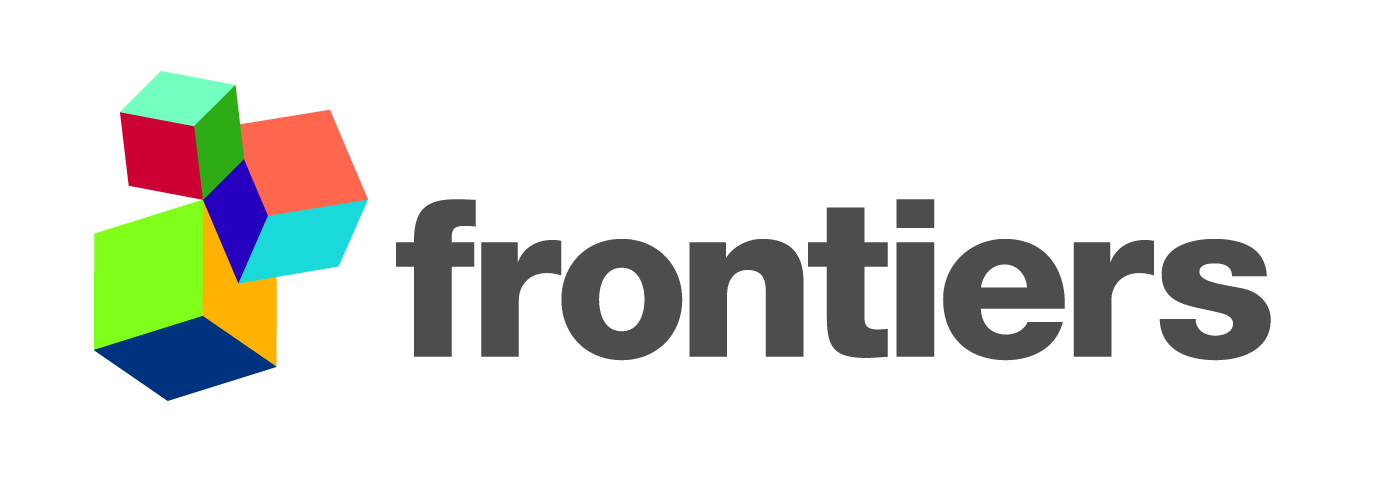

Supplement: Supplementary file 1 [file Data_Sheet_1.DOCX]
